# Sindbis viral structural protein cytotoxicity on human neuroblastoma cells

**DOI:** 10.1007/s00383-020-04719-8

**Published:** 2020-07-21

**Authors:** Eriko Y. Saito, Kengo Saito, Tomoro Hishiki, Ayako Takenouchi, Takeshi Saito, Yoshiharu Sato, Keita Terui, Tadashi Matsunaga, Hiroshi Shirasawa, Hideo Yoshida

**Affiliations:** 1 Department of Pediatric Surgery, Graduate School of Medicine, Chiba University, 1-8-1 Inohana, Chuou-ku, Chiba, 260-8670 Japan; 2 Molecular Virology, Graduate School of Medicine, Chiba University, 1-8-1 Inohana, Chuou-ku, Chiba, 260-8670 Japan

**Keywords:** Apoptosis, E1, Neuroblastoma, UV, Sindbis virus

## Abstract

**Purpose:**

Oncolytic viral therapy for neuroblastoma (NB) cells with Sindbis virus (SINV) is a promising strategy for treating high-risk NB. Here, we evaluated the possibility of using SINV structural proteins as therapeutic agents for NB since UV-inactivated SINV could induce cytopathogenic effects.

**Methods:**

The cytotoxicity of UV-inactivated SINV toward human NB cell lines NB69, NGP, GOTO, NLF, SK-N-SH, SH-SY5Y, CHP134, NB-1, IMR32, and RT-BM-1 were analyzed. Apoptosis was confirmed by TUNEL assays. To determine the components of SINV responsible for the cytotoxicity of UV-inactivated SINV, expression vectors encoding the structural proteins, namely capsid, E2, and E1, were transfected in NB cells. Cytotoxicity was evaluated by MTT assays.

**Results:**

UV-inactivated SINV elicited more significant cytotoxicity in NB69, NGP, and RT-BM-1 than in normal human fibroblasts. Results of the transfection experiments showed that all NB cell lines susceptible to UV-inactivated SINV were highly susceptible to the E1 protein, whereas fibroblasts transfected with vectors harboring capsid, E1, or E2 were not.

**Conclusions:**

We demonstrated that the cytotoxicity of the UV-inactivated SINV is due to apoptosis induced by the E1 structural protein of SINV, which can be used selectively as a therapeutic agent for NB.

## Introduction

Neuroblastoma (NB) is the most common solid extracranial tumor of early childhood [1, 2]. NB patients with high-risk disease continue to exhibit poor prognosis even after intensive chemotherapy and autologous bone marrow transplantation [3–5], which limits their treatment options. The resistance of NBs to conventional therapies has prompted the search for a novel therapeutic approach based on the naturally occurring oncolytic virus, Sindbis virus (SINV).

Previously, we have reported that the SINV AR339 strain may be used as a novel therapeutic agent for human cervical, ovarian, and oral cancer [6–8]. Human NB cells and NB xenograft tumors are also susceptible to replication-competent SINV [9]. SINV, a mosquito-borne alphavirus, is an enveloped, plus-strand RNA virus that causes infection of neurons and age-dependent encephalomyelitis in mice, and mild mosquito-borne viral arthritis in humans [10].

Neurons are the primary targets for SINV infection in the central nervous system (CNS), and both host and viral factors determine the fate of the infected neurons [11]. However, alphaviruses can also cause persistent infection, which has been clearly shown for mosquito cells and differentiated mammalian neurons [12, 13]. SINV, similar to other alphaviruses, encodes four nonstructural proteins (nsPs) and three major structural proteins—a capsid protein and two surface glycoproteins, E1 and E2 [14]. E1 and E2 heterodimerize and then trimerize to form spikes on the virion surface that mediate virus binding and entry. While a role for nsPs in the establishment of persistent SINV infection has been suggested [15], infection of cells with SINV replicons has shown that the structural proteins dramatically accelerate cell death [16].

UV-inactivated SINV can induce apoptosis in infected cells, and replication-competent SINV can induce apoptosis at the time of fusion with the cell membrane with no viral replication [17, 18]. E2 is the primary determinant of binding to cellular receptors, and E1 is required to promote the fusion between viral particles and cell membranes [19]. However, the mechanism via which these structural proteins cause cell death is not well understood.

In this study, we investigated the cytotoxic activity of the UV-inactivated SINV against human NB cells and evaluated the potential of structural proteins of SINV as possible therapeutic agents in NB.

## Materials and methods

### Cells

Human NB cell lines NB69 (ECACC Cat# 99072802, RRID:CVCL_1448), NGP (RRID:CVCL_AX38), GOTO (JCRB Cat# JCRB0612.1, RRID:CVCL_2911), NLF (RRID:CVCL_UJ92), SK-N-SH (RRID:CVCL_D044), SH-SY5Y (RRID:CVCL_YK67), CHP134 (RCB Cat# RCB0487, RRID:CVCL_1124), NB-1 (RCB Cat# RCB1953, RRID:CVCL_1440), IMR32 (CLS Cat# 300148/p666_IMR-32, RRID:CVCL_0346), and RT-BM-1 (JCRB Cat# IFO50432, RRID:CVCL_1339) were obtained from the Japanese Cancer Research Resources Bank (Tokyo). Laboratory stocks of Vero (CLS Cat# 605372/p622_VERO, RRID:CVCL_0059) cells [20] and human fibroblasts derived from gingiva were also used. Human NB cell lines were grown in Roswell Park Memorial Institute (RPMI) 1640 medium supplemented with 10% heat-inactivated fetal bovine serum (FBS) and 100 μg/mL kanamycin (Sigma, St. Louis, MO, USA) at 37 °C in an atmosphere of 5% CO_2_. Vero cells and human fibroblasts were maintained in Dulbecco’s modified Eagle’s medium (DMEM) supplemented with 10% FBS at 37 °C in an atmosphere of 5% CO_2_.

### Viruses

SINV AR339 was provided by the National Institute of Infectious Diseases (Tokyo, Japan). It was propagated in primary chicken embryo fibroblasts and then passaged several times in Vero cells. SINV titer was determined using plaque assays with Vero cells. SINV was inactivated using UV-irradiation for 45 min. Plaque assays confirmed the inactivation of SINV.

### MTT assay

Cells were plated on 24-well plates at the density of 1 × 10^5^ cells/well and infected with SINV at a multiplicity of infection (MOI) of 1. Cells were also infected with UV-inactivated SINV at an MOI of 1 or 5. Cell viability was evaluated after 48 h. Each plate was equilibrated to room temperature (approximately 25 °C) for 20 min followed by the addition of 200 μL CellTiter Glo reagent (Promega, Madison, WI, USA) to each well. Plates were gently agitated on a plate shaker for 1 min and incubated for an additional 10 min at room temperature followed by immediate evaluation of cell viability by measuring the luminescence using a plate reader (Wallac 1420 ARVOsx Multilabel Counter, Perkin-Elmer, Japan). The cell viability of mock infection was considered 100%. These data were presented as mean ± standard deviation (SD) of three determinations.

### Terminal deoxynucleotide transferase-mediated dUTP nick-end labeling (TUNEL) assay

Cancer cells were seeded at the density of 1 × 10^5^ cells/well on Lab-Tek chamber slides (Thermo Fisher Scientific, Japan). The following day, the cells were treated with UV-inactivated SINV at an MOI of 5. Twenty-four hours after adsorption of UV-inactivated SINV, the cells were washed twice with phosphate-buffered saline (PBS) and fixed with 4% paraformaldehyde for 15 min. TUNEL assay was performed according to the protocol of an in situ apoptosis detection kit (Takara Bio, Japan).

### Expression vectors

The cDNAs of SINV C (nt 7647-8449), 6KE1 (nt 9900-11474), E3E2 (nt 8449-9900), 6kE1ΔTMD (nt 9900-11283), E1ΔFD (nt 10693-11474), and 6K (nt 9900-10064) with *Eco*RI adapter sequences were obtained via reverse transcription-polymerase chain reaction (RT-PCR) of the SINV genomic RNA. The forward and reverse primers were 5′-ACCGAATTCATGAATAGAGGATTC-3′ and 5′-GGTCTAGATCACCACTCTTCTGTCC-3′ for C; 5′-CGGAATTCATGGAAACGTTCACCGAGACC-3′ and 5′-CGTCTAGATCATCATCTCGTGTGCTAGT-3′ for 6KE1; 5′-ACAGAATTCATGTCCGCAGCACCAC-3′ and 5′- GGTTCTAGATCAAGCATTGGCCGAC-3′ for E3E2; 5′-ATGGAAACGTTCACCGAGACCATGAGTTAC-3′ and 5′-TCATGATGTTTTTGAGATGGCGGCTTGAAA-3′ for 6kE1ΔTMD; 5′-ATGAGCAAGGATCTCATCGCCAGCACAGAC-3′ and 5′-TCATCATCTCGTGTGCTAGT-3′ for E1ΔFD; and 5′-ATGGAAACGTTCACCGAGACCATGAGTTAC-3′ and 5′-TCAGGTGTCTACCTTCGCCAGGTAGGCGCC-3′ for 6K. After purification, the PCR products and the expression vector pCI-neo (Promega, USA) were digested with *Eco*RI (Takara Bio, Japan) and ligated using T4 DNA ligase (Takara Bio, Japan).

### Transfection of expression vectors

NB69 cells were seeded on a six-well plate at the density of 2 × 10^5^ cells/well and cultured overnight at 37 °C in RPMI supplemented with 10% FBS. One microgram of each vector DNA was added and diluted in 500 μL RPMI supplemented with 10% FBS. The diluted solution was mixed with Lipofectamine LTX reagent (Invitrogen, Japan) and incubated at room temperature for 30 min to form a complex. The DNA–Lipofectamine LTX complex was added to the plate, and the cells were cultured for 4 h. The medium was replaced with fresh medium, and the cells were further cultured for 18–24 h. For selecting transfectants, the cells were cultured in a medium containing 300 μg/mL neomycin for 96 h. Cell viability assays were subsequently performed.

### Statistical analysis

All values are expressed as mean ± SD. Statistical analyses were performed using *t* tests with Welch’s correction. A *p* value (*p*) < 0.01 was considered to be statistically significant.

## Results

### Cytopathic effects of UV-inactivated SINV

**Fig. 1:**
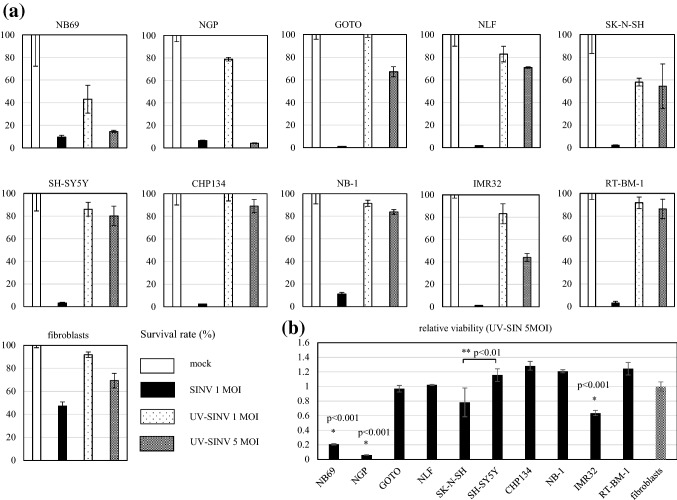
Cytopathic effects of UV-inactivated Sindbis virus. Human neuroblastoma cell lines NB69, NGP, GOTO, NLF, SK-N-SH, SH-SY5Y, CHP134, NB-1, IMR32, and RT-BM-1, as well as the primary cultured normal human fibroblasts, were treated with SINV at an MOI of 1 (SINV 1 MOI) and UV-inactivated SINV at an MOI of 1 and 5 (UV-SINV 1 MOI and 5 MOI). **a** Cell viabilities 48 h after treatment are shown as percentages of mock infection, which was considered to be 100%. **b** Relative viabilities of neuroblastoma cell lines treated with UV-inactivated SINV at an MOI of 5 are shown relative to that of normal human fibroblasts, which was considered 1.0. All values are expressed as mean ± standard deviation (SD). Statistical analyses were performed using *t* tests with Welch’s correction. *Significance of differences between NB69 and fibroblasts, NGP and fibroblasts, and IMR32 and fibroblasts. **Significance of the difference between SK-N-SH and SH-SY5Y cells

In a previous preliminary study, we found that UV-inactivated SINV was cytotoxic to NB cells (unpublished data). To determine whether the structural proteins alone of SINV can induce apoptosis, we treated human NB cell lines (NB69, NGP, GOTO, NLF, SK-N-SH, SH-SY5Y, CHP134, NB-1, IMR32, and RT-BM-1) as well as a primary cultured normal human fibroblast line with UV-inactivated SINV at an MOI of 1 and 5. Theoretically, infection of cells with viruses at an MOI of 1 and 5 results in the adsorption of viral particles to 63% and 99% of the cells, respectively. Based on these observations, cells in this study were treated with SINV at an MOI of 1. Cell viability assays showed that UV-inactivated SINV was cytotoxic for NB cells after 48 h, and elicited evident effects on NB69 and NGP cells in a dose-dependent manner; however, the levels of cytotoxicity induced by UV-inactivated SINV was lower than that induced by replication-competent SINV (Fig. 1a). Compared to that observed in fibroblasts, UV-inactivated SINV at an MOI of five showed significant cytotoxicity toward NB69, NGP, and IMR32 cells, but not on other NB cells (Fig. 1b). The cell line SK-N-SH was more susceptible to UV-inactivated SINV than SH-SY5Y, which is a subclone of SK-N-SH. The SK-N-SH cell line consists of S cells and N cells, whereas the SH-SY5Y cell line consists of only N cells [21]. Therefore, the phenotypes of S cells might be related to the susceptibility of the SK-N-SH cell line to the cytotoxic properties of UV-inactivated SINV.

### UV-inactivated SINV induced apoptosis of neuroblastoma cells

**Fig. 2:**
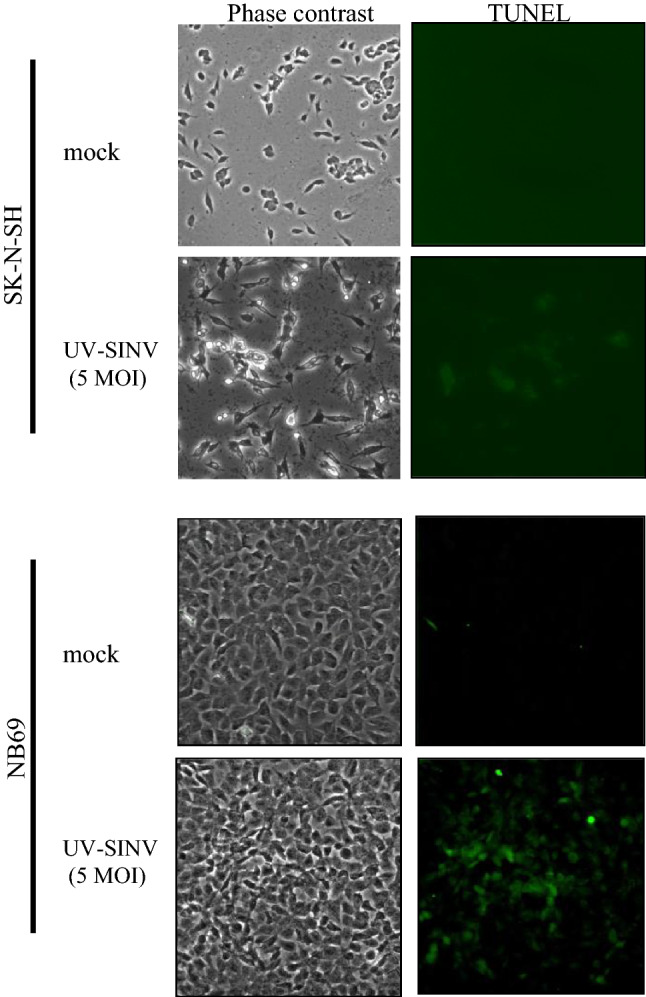
TUNEL assays of SK-N-SH and N69 cells 18 h after treatment with UV-inactivated SINV at an MOI of 5. SK-N-SH and NB69 cells were treated with UV-inactivated SINV at an MOI of 5 (UV-SINV) or an equal volume of medium (mock). Micrographs of phase contrast and TUNEL assays 18 h after treatment of SK-N-SH and NB69 cells are shown

To determine whether the cytotoxicity is due to the induction of apoptosis, TUNEL assays were performed 18 h after treating SK-N-SH and NB69 cells with UV-inactivated SINV at an MOI of 5. As shown in Fig. 2, TUNEL-positive cells were observed in both cell lines but were more intense in NB69 cells. The relative signal intensities were in agreement with the cytotoxicity of UV-inactivated SINV toward these cells.

### Cytotoxicity of SINV C, E1, and E2 toward NB cells

**Fig. 3:**
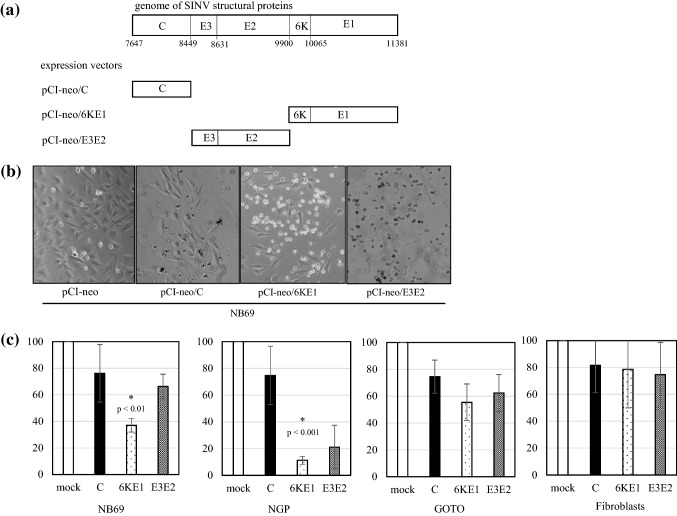
Cytotoxicities of SINV structural proteins. **a** The genome of SINV structural proteins and the corresponding sequences expressed from the expression vectors pCI-neo/C, pCI-neo/E2E2, and pCI-neo/6KE1. **b** Phase-contrast micrographs of NB69 cells transfected with expression vectors pCI-neo (mock), pCI-neo/C, pCI-neo/6KE1, and pCI-neo/E3E2. **c** Viabilities of NB69, NGP, GOTO, and fibroblasts transfected with pCI-neo (mock), pCI-neo/C (C), pCI-neo/6KE1 (6KE1), and pCI-neo/E3E2 (E3E2) are shown as percentages of mock transfection, which was considered to be 100%. All values are expressed as mean ± standard deviation (SD). Statistical analyses were performed using *t *tests with Welch’s correction. *Significance of the difference between 6KE1 and mock

As UV-inactivated SINV is unable to replicate in infected cells, the structural components of SINV that are not involved in viral replication were believed to be responsible for its cytotoxicity. To identify the viral components responsible for the induction of apoptosis, we constructed the expression vectors pCI-neo/C, pCI-neo/E3E2, and pCI-neo/6KE1, which encoded the viral structural proteins C, E3E2, and 6KE1, respectively (Fig. 3a). E3, which is not present in the viral particle, is necessary for proper processing of E2 and is cleaved before viral particle assembly. 6K is a minor component of the viral particle and is cleaved from the E1 protein before viral particle assembly [14].

NB69 and NGP cells, which are susceptible to UV-inactivated SINV and less susceptible than GOTO cells, and fibroblasts were transfected with vectors expressing C, 6KE1, or E3E2, and selected using neomycin. As shown in Fig. 3b, the most significant cytotoxicity was observed when 6KE1 was expressed. 3-(4,5-Dimethylthiazol-2-yl)-2,5-diphenyltetrazolium bromide (MTT) assays were used to assess cytotoxicity (Fig. 3c). The results of the MTT assays showed that NB69 and NGP cells were significantly susceptible to 6KE1, while fibroblasts transfected with an empty vector (mock) or C, 6KE1, or E3E2 remained viable.

### Cytotoxicity of SINV 6KE1 deletion mutants toward neuroblastoma cells

**Fig. 4:**
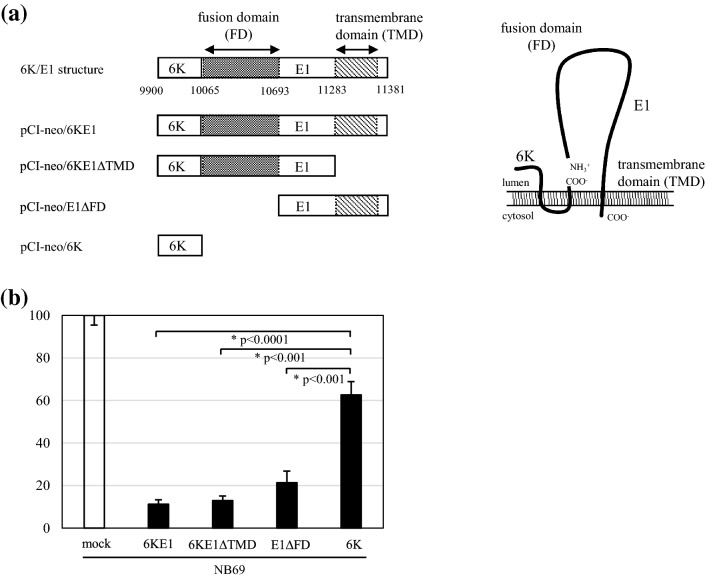
Cytotoxicities of SINV 6K/E1 deletion mutants. **a** Map of SINV 6K/E1 genes and the corresponding sequences expressed in the expression vectors pCI-neo/6KE1, pCI-neo/6KE1ΔTMD, pCI-neo/E1ΔFD, and pCI-neo/6K. Localization of SINV 6K, fusion domain (FD), and transmembrane domain (TMD) of the E1 protein in the endoplasmic reticulum are depicted in the right panel. **b** Viabilities of NB69 cells transfected with pCI-neo (mock), pCI-neo/6KE1 (6KE1), pCI-neo/6KE1ΔTMD (6KE1ΔTMD), pCI-neo/E1ΔFD, and pCI-neo/6K (6K) are shown as percentages of mock transfection, which was considered to be 100%. All values are expressed as mean ± standard deviation (SD). Statistical analyses were performed using *t* tests with Welch’s correction. *Significance of difference between 6KE1 and 6K (*p* < 0.0001), 6KE1ΔTMD and 6K (*p* < 0.001), and E1ΔFD and 6K (*p* < 0.001)

As 6KE1 was more cytotoxic to NB cells than C and E3E2, we attempted to identify the domain of 6KE1 responsible for cytotoxicity using 6KE1 deletion mutants. The N terminus of E1 is a fusion domain (FD) vital for the fusion of the virus to the host cell surface, while the C terminus is a transmembrane domain (TMD) anchored to the endoplasmic reticulum [14]. We constructed pCI-neo/6KE1ΔTMD lacking TMD, pCI-neo/E1ΔFD lacking 6K and FD, and pCI-neo/6K expressing only 6K for this purpose (Fig. 4a).

As shown in Fig. 4b, 6KE1, 6KE1ΔTMD, and E1ΔFD were significantly more cytotoxic to NB69 cells than 6K. The 6K deletion mutants lacking FD or TMD were still cytotoxic, suggesting that domains other than FD and TMD were involved in the cytotoxic activity of E1.

## Discussion

Virotherapy for cancer is a promising strategy. However, one issue hindering its clinical application is safety [8]. To enhance safety, we tested the possibility of selectively utilizing specific viral components to induce cell death. To explore the possibility of using structural proteins of SINV as a therapeutic agent for neuroblastoma, we examined their cytotoxicity toward human neuroblastoma cells and found some neuroblastoma cells to be highly susceptible to the SINV structural protein E1.

SINV infects neurons and induces apoptosis, showing cytotoxicity toward human neuroblastoma cells [8, 9]. Previous studies have shown that SINV-induced apoptosis is most efficiently triggered in the presence of structural proteins [16, 22], and transient overexpression of SINV structural proteins or UV-inactivated SINV can induce apoptosis in Chinese hamster ovary (CHO) cells and N18 mouse neuroblastoma cells [17, 18]. In this study, we reported that UV-inactivated SINV induced apoptosis in several human NB cell lines.

NB cells have evolved redundant strategies for avoiding apoptosis, which determine the resistance of this tumor to treatment. For example, down-regulation of TRAIL or CD95 receptors, or enhanced expression of anti-apoptotic proteins, such as survivin or Bcl-2, is observed in NB cells [16, 22, 23]. SINV induces apoptosis in most vertebrate cell lines and can be blocked in cells where anti-apoptotic Bcl-2 is expressed [12]. Our previous reports have suggested that human NB cells highly expressing Bcl-2 are resistant to SINV-induced cytotoxicity [7, 9]. Thus, cell death signals may be blocked by multiple mechanisms in NB cells, which may, at least partially, compensate for each other. Therefore, the variable susceptibility of NB cells to UV-inactivated SINV might be due to the redundancy of apoptosis in NB cells.

The SH-SY5Y cell line is an N-type subclone of SK-N-SH consisting of both N and S cell types [21], for which the susceptibilities to UV-inactivated SINV vary, indicating that the susceptibility to UV-inactivated SINV might be related to the NB cell types. N-cells show characteristics of neuronal cells and generally express high levels of Bcl-2, while S-cells show an epithelioid phenotype and express lower levels of Bcl-2 [22]. However, NGP cells, which were highly susceptible to UV-inactivated SINV, consisted of typical N-type cells. Therefore, the susceptibility of NB cells to UV-inactivated SINV might not be related only to the N- and S-types of NB cells. Acquired genomic alterations, including MYCN amplification and 1p alteration, are important risk factors for neuroblastoma [1, 2]. Common characteristics of NB69, NGP, and IMR32 cell lines that were susceptible to UV-inactivated SINV, suggested 1p alterations [24]. As 1p alterations are also found in the less susceptible cell lines NLF, CHP134, and NB-1, the sensitivity to SINV structural proteins does not seem to correlate directly to the 1p genomic alteration. Although this study could not identify the common characteristics of the three cell lines that correlated with sensitivity, a subpopulation of neuroblastoma cells should be responsive to SINV structural proteins.

E1 and E2 are the main SINV structural proteins. E2 allows SINV to adhere to the viral receptor on the cell surface, and E1 is known to possess cell membrane fusion activity [25]. Viral replication is not necessarily required for SINV-induced apoptosis, and even UV-inactivated SINV can induce apoptosis [17, 18]. In addition, the E1 and E2 structural proteins of SINV possess cytotoxic properties,furthermore, E1 and E2 heterodimers or E1 alone exhibit cytotoxicity, although E2 alone is not strongly cytotoxic [16, 19]. Our study indicated that E1 induced higher cytotoxicity in NB cells than E2.

The cytotoxicity of E1 is possibly due to its cell membrane fusion activity. The sphingomyelin-dependent fusion of the viral envelope with the endosomal membranes initiates apoptosis by inducing sphingomyelin degradation and ceramide release [17]. The plasma membranes of eukaryotic cells consist primarily of sphingolipids, glycerophospholipids, and cholesterol [26]. Sphingomyelin, consisting of a highly hydrophobic ceramide moiety and a hydrophilic phosphorylcholine head group, is the most prevalent cellular sphingolipid and is particularly abundant in the nervous system [26, 27]. Ceramide is implicated as a mediator in the signaling pathway of various inducers of apoptosis, including tumor necrosis factor (TNF), Fas ligand, neurotrophins, ionizing radiation, cytokines, heat shock, UV light, antitumor drugs, and oxidative stress [26, 28–31].

Although virotherapy that uses replication-competent viruses with less pathogenicity and higher specificity to cancer, such as SINV, should be a promising strategy, the possibility that the virus could revert or evolve into a pathogen as it propagates in the patient is a concern [8]. The selective application of the viral component that elicits cancer-specific cytotoxicity as an anti-cancer agent would be an effective strategy to improve safety. In this study, we confirmed the cytotoxic activity of UV-inactivated SINV against human NB cells. We demonstrated that the cytotoxicity of the SINV structural protein against NB cells may be attributed to apoptosis induced by the E1 protein of SINV. Using viral components for virotherapy may circumvent the issues involved with the use of replication-competent viruses. Further analyses of the cytotoxicity of SINV E1 toward NB may provide an alternative strategy for combination therapies using other anti-cancer drugs.
